# Worldwide trend analysis of primary and secondary infertility rates over past decades: A cross-sectional study

**DOI:** 10.18502/ijrm.v20i1.10407

**Published:** 2022-02-18

**Authors:** Nasrin Borumandnia, Hamid Alavi Majd, Naghmeh Khadembashi, Hojat Alaii

**Affiliations:** ^1^Urology and Nephrology Research Centre, Shahid Beheshti University of Medical Sciences, Tehran, Iran.; ^2^Department of Biostatistics, School of Allied Medical Sciences, Shahid Beheshti University of Medical Sciences, Tehran, Iran.; ^3^Department of Foreign Languages Education, School of Allied Medical Sciences, Shahid Beheshti University of Medical Sciences, Tehran, Iran.; ^4^Department of Biostatistics, School of Allied Medical Sciences, Shahid Beheshti University of Medical Sciences, Tehran, Iran.

**Keywords:** Infertility, Global burden of disease, Longitudinal studies.

## Abstract

**Background:**

Infertility is a global health issue and is reported differently worldwide.

**Objective:**

To assess the longitudinal trends of primary and secondary infertility prevalence rate (PSIPR) per 100,000 across all countries during past decades.

**Materials and Methods:**

The PSIPR was extracted from the Global Burden of Disease database for 195 countries during 1993-2017. The longitudinal trends of PSIPR were explored across the seven epidemiological regions designated by the Global Burden of Disease.

**Results:**

Globally, the PSIPR was lower among men than women. Over time, the prevalence of primary infertility in men and women had a decreasing trend of -9.3 and -11.6 in high-income countries. Other regions have seen an increase, the highest being in South Asian women, and men of the Middle East and North Africa, with rates of change of 40.9 and 19.0, respectively. Over time, the secondary infertility prevalence in women of Central Asia, Central Europe and Eastern Europe, as well as of high-income countries, has been declining (rates of change of -16.9 and -11.7, respectively). Other regions have been on the rise, with the highest increase among women of the Middle East, North Africa, and South Asia (trend of 119.9 and 83.4, respectively), and in South Asian men (trend of 48.4).

**Conclusion:**

The overall trend of infertility prevalence shows a downward trajectory in high-income and developed countries and an upward trend in others. These findings might be explained by missed cases of infertility due to a low tendency for reproduction and the presence of more infertility treatment facilities in these regions.

## 1. Introduction

Primary infertility is considered as occurring in a couple who have not had a live birth for more than five yr while they have been in a relationship without contraceptives. A couple with the desire to have a child who have been in a relationship for more than five yr without using contraceptives, and without a live birth since a previous live birth are referred to as having secondary infertility (1).

Infertility is a global public health issue with a high impact on individuals of both sexes and society. Infertility is ranked as the 5 ${}^{\mathrm{th}}$ highest serious global disability with a negative impact on the self-esteem of those who are involved. These negative side effects remain a higher social burden for women than men (2).

More than half of infertility occurs in men (3). According to reports, some areas such as North Africa and the Middle East have witnessed a high rate of primary infertility prevalence, while secondary infertility has had a low prevalence. On the other hand, other regions such as Central and Western Europe have witnessed the opposite (4). The global prevalence of primary infertility and secondary infertility during 1990 to 2010 were reported in one study to be between 0.6-3.4% and 8.7-32.6%, respectively (5). Another study estimated the primary infertility prevalence to be within the range of 1.5-2.6%, which was lower than secondary infertility (7.2-18%) in 2009-2010 (6). According to another study, approximately 10.5% (with a range from less than 6% to greater than 16% by region and country) and 2% of women have experienced primary and secondary infertility, respectively, across the world during 1982-2010 (7). Infertility rates have been reported to be different in developed nations comparing with non-developed ones. Based on a meta-analysis of population surveys published since 1990, the infertility prevalence was estimated to range from 3.5-16.7% in developed regions and from 6.9-9.3% in developing nations (8). There are sparse data about the primary and secondary infertility prevalence in different regions of the world, and this statistic does not accurately represent infertility across regions in the world. In other words, there is no single outbreak, and great variations exist within and across different countries in each continent and region. Few comparative analyses have been conducted to explore the trends of primary and secondary infertility rates regionally and globally in both sexes over multiple decades.

Therefore, this study was designed to investigate the trend of primary and secondary infertility prevalence rates over the past three decades, in the various epidemiological regions of the world. To achieve this goal, longitudinal data of primary and secondary infertility prevalence rates in previous years were compiled from the global burden of disease (GBD) database across the epidemiological sub-regions designated by the GBD.

## 2. Materials and Methods 

### Data source

This was a cross-sectional study, in which longitudinal data from 1993-2017 for prevalence rates of primary and secondary infertility (per 100,000 people) for 195 countries and territories were extracted from the GBD database, from the Institute for Health Metrics and Evaluation. The GBD is the most comprehensive observational epidemiological study which reports the prevalence, incidence, disability-adjusted life-years, death, etc. for diseases, and risk factors for all countries worldwide (9). The information used in this study included the prevalence rates of both primary and secondary infertility for males and females from 1993 to 2017. Seven regions were considered, including `High Income', `Central Asia and Central and Eastern Europe', `Latin America and Caribbean', `North Africa and Middle East', `South-East Asia, East Asia and Oceania', `South Asia', and `Sub-Saharan Africa'. The mentioned regions were designated by the GDB study. The trend of primary and secondary infertility prevalence rates was assessed in the regions, separately in males and females.

### Ethical considerations

The Ethics Committee of Shahid Beheshti University of Medical Sciences approved this study (Code: IR.SBMU.RETECH.REC.1398.099).

### Statistical analysis

The prevalence of primary and secondary infertility in each region and year (at six-year intervals, which was considered due to statistical modelling and data structure considerations) were described together with the mean and standard deviation. The latent growth method (LGM) was used to estimate the change in prevalence rate of primary and secondary infertility in different regions, separately in males and females. The growth trajectory of prevalence rates over time was also estimated through LGM methods. The LGM regression coefficients showed the rate of change in prevalence over time (10). The statistical analysis was performed using the M-plus version 6.12 (www.statmodel.com).

## 3. Results

**Figure 1:**
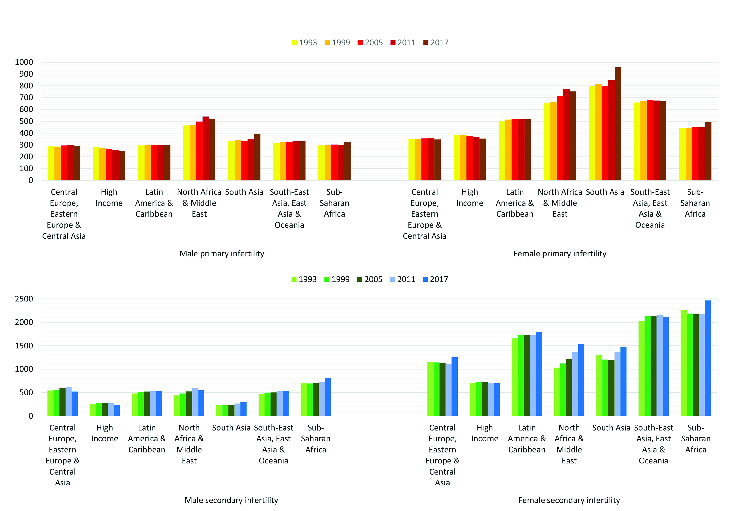
The means of infertility rates per 100,000 people in various regions during 1993-2017.

The mean and standard deviation of the primary and secondary infertility prevalence rates in seven regions are reported in tables I and II, respectively. The plots in figure 1 show the mean rates of infertility in various regions over this period. Also, the regression coefficients estimated from the LGM are presented in the last column of the tables. The intercept coefficients in the table show the estimated mean of the initial infertility rate in 1993. The average change in the infertility rate over time in each region is reported as the slope. The sign of the slope coefficient shows that the infertility rate had an increasing (positive sign) or decreasing (negative sign) trend over the period. For instance, the estimate of primary infertility of females in High-Income countries (intercept = 398.1, slope = -11.6) revealed that the initial prevalence rate of infertility was estimated to be 398.1 per 100,000 people in 1993 and it had a decreasing trend of -11.6 per six-year period until 2017. For another example, the LGM result for North Africa and Middle East females indicated that the initial prevalence rate of infertility was 630.3 per 100,000 people and it had an increasing trend with a slope of about 38.5 until 2017.

The results of table I showed that in all areas, the primary infertility prevalence rate at the beginning of the study period was slightly lower among men than women. Also, the prevalence of primary infertility in men and women in High-Income countries has had a decreasing trend, with an average rate of change of -9.3 and -11.6 per 100,000 people, respectively. Other regions have seen an increase in the prevalence of primary infertility. South Asian women had the highest average rate of change (40.9 per 100,000), followed by females in North Africa and the Middle East (with an average rate of change of 38.5 per 100,000). Regarding primary infertility in men, North Africa and the Middle East (with a rate of change of 19.0 per 100,000) and South Asia (with an average rate of change of 16.5 per 100,000) had the highest increasing rates.

**Figure 2:**
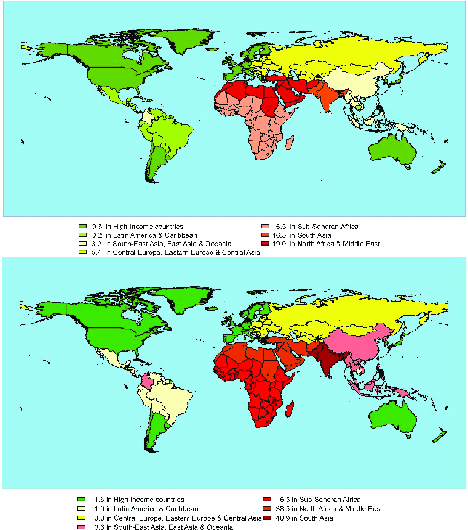
World maps based on changes in primary infertility rates during 1993-2017. The average rates of change in male (top) and female (bottom) primary infertility prevalence per 100,000 people.

**Figure 3:**
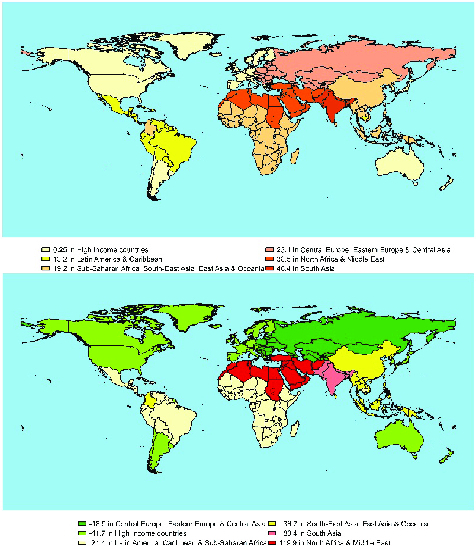
World map based on changes in secondary infertility rates during 1993-2017. The average rate of change in male (top) and female (bottom) secondary infertility prevalence per 100,000 people.

According to the results of secondary infertility shown in table II, once more, in all regions, the prevalence of secondary infertility at the beginning of the study period was higher among females compared with men. Over time, the rate of secondary infertility prevalence in women in Eastern and Central Europe, and Central Asia, as well as in High-Income countries have declined with an average rate of change of -16.9 and -11.7 (per 100,000 people), respectively. Other regions have been on the rise, with the highest increase in North Africa and the Middle East among women (average rate of change: 119.9 per 100,000), followed by South Asian women (average rate of change: 83.4 per 100,000) and women in South-East Asia and Oceania (average rate of change: 36.7 per 100,000). Regarding secondary infertility in males, the prevalence rates have been increasing in all regions. The highest increase was found in South Asian men (with an average rate of change of 48.4 per 100,000) and the lowest increase was observed among men in High-Income regions (with an average rate of change of 0.25 per 100,000). The maps in figures 2 and 3 reveal the estimated trend of primary and secondary infertility rates from 1993 up to 2017 in the GBD regions.

**Table 1:** Primary infertility prevalence rates per 100,000 people and the latent growth model results for analysis of trends


** GBD regions**		**1993**	**1999**	**2005**	**2011**	**2017**	**LGM estimates**
**Central Europe, Eastern Europe & Central Asia**							
	**Female**	348.8 ± 115.9	347.9 ± 113.9	354.9 ± 120.2	355.6 ± 124.0	347.3 ± 125.3	Intercept: 348.3 Slope: 3.0 (p = 0.15)
	**Male**	287.1 ± 100.0	285.7 ± 98.2	295.3 ± 107.6	299.9 ± 112.7	291.9 ± 111.9	Intercept: 248.9 Slope: 5.4 (p < 0.001)
**High income**							
	**Female**	387.7 ± 142.1	387.8 ± 142.9	387.8 ± 142.9	365.6 ± 135.5	353.7 ± 129.0	Intercept: 398.1 Slope: -11.6 (p < 0.001)
	**Male**	280.1 ± 98.4	276.2 ± 95.3	276.2 ± 95.3	258.6 ± 90.2	250.7 ± 86.5	Intercept: 284.8 Slope: -9.3 (p < 0.001)
**Latin America & Caribbean**							
	**Female**	501.9 ± 235.9	513.5 ± 239.2	518.9 ± 231.1	518.2 ± 217.8	516.9 ± 206.3	Intercept: 510.8 Slope: 1.9 (p = 0.66)
	**Male**	295.1 ± 144.4	298.9 ± 144.0	299.7 ± 137.4	298.6 ± 128.1	298.7 ± 121.2	Intercept: 298.7 Slope: -0.2 (p = 0.92)
**North Africa & Middle East**							
	**Female**	652.5 ± 109.8	665.5 ± 123.7	715.8 ± 146.4	774.6 ± 152.8	753.3 ± 144.2	Intercept: 630.3 Slope: 38.5 (p < 0.001)
	**Male**	465.4 ± 100.8	468.5 ± 111.7	497.8 ± 123.7	540.4 ± 140.9	518.1 ± 127.3	Intercept: 456.8 Slope: 19.0 (p < 0.001)
**South Asia**							
	**Female**	798.4 ± 197.4	816.9 ± 240.8	799.1 ± 221.4	848.8 ± 263.6	960.4 ± 254.5	Intercept: 775.1 Slope: 40.9 (p < 0.001)
	**Male**	330.6 ± 90.0	338.3 ± 107.0	331.2 ± 101.7	349.8 ± 116.5	390.9 ± 109.0	Intercept: 321 Slope: 16.5 (p < 0.001)
**South-East Asia, East Asia & Oceania**							
	**Female**	657.7 ± 258.6	674.3 ± 261.3	681.0 ± 267.2	676.4 ± 267.8	672.0 ± 263.9	Intercept: 666.0 Slope: 3.6 (p = 0.54)
	**Male**	318.7 ± 125.9	324.8 ± 125.8	327.8 ± 126.6	330.1 ± 129.9	331.6 ± 129.4	Intercept: 320.2 Slope: 3.2 (p = 0.31)
**Sub-Saharan Africa**							
	**Female**	446.3 ± 177.8	445.3 ± 189.3	453.4 ± 228.6	450.7 ± 243.8	493.1 ± 247.5	Intercept: 442.0 Slope: 6.5 (p = 0.23)
	**Male**	294.8 ± 131.5	298.0 ± 142.8	304.2 ± 159.7	298.0 ± 155.2	326.4 ± 168.9	Intercept: 294.5 Slope: 3.5 (p = 0.33)
Data shown as Mean ± SD. The intercepts assess the estimated overall mean level of primary infertility rate in 1993. The slopes are interpreted as the average change in primary infertility rate over time. GBD: Global burden of disease, LGM: Latent growth method							

**Table 2:** Secondary infertility prevalence rates per 100,000 people and the latent growth model results for analysis of trends


** GBD region**		**1993**	**1999**	**2005**	**2011**	**2017**	**LGM estimates**
**Central Europe, Eastern Europe & Central Asia**							
	**Female**	1147.0 ± 197.3	1143.8 ± 212.2	1138.7 ± 209.1	1111.6 ± 203.6	1253.7 ± 218.2	Intercept: 1150.0 Slope: -16.9 (p = 0.07)
	**Male**	546.0 ± 99.9	561.2 ± 110.4	592.5 ± 117.2	612.5 ± 120.3	520.7 ± 94.6	Intercept: 545.6 Slope: 23.1 (p < 0.001)
**High Income**							
	**Female**	709.5 ± 261.6	732.2 ± 299.6	723.8 ± 299.9	695.6 ± 275.8	701.9 ± 271.4	Intercept: 741.6 Slope: -11.7 (p < 0.001)
	**Male**	262.4 ± 112.9	279.7 ± 129.9	284.1 ± 128.9	278.3 ± 120.9	242.0 ± 98.9	Intercept: 277.1 Slope: 0.3 (p = 0.92)
**Latin America & Caribbean**							
	**Female**	1659.4 ± 659.1	1734.0 ± 707.2	1735.4 ± 639.3	1723.2 ± 567.4	1797.2 ± 551.9	Intercept: 1687.4 Slope: 24.4 (p = 0.06)
	**Male**	479.9 ± 206.6	508.7 ± 225.7	520.8 ± 211.2	529.0 ± 187.3	540.3 ± 168.4	Intercept: 491.4 Slope: 13.2 (p < 0.001)
**North Africa & Middle East**							
	**Female**	1031.7 ± 329.4	1122.0 ± 383.1	1214.6 ± 396.0	1365.3 ± 408.1	1544.2 ± 451.3	Intercept: 1011.0 Slope: 119.9 (p < 0.001)
	**Male**	439.9 ± 214.7	480.2 ± 233.6	523.5 ± 239.7	603.9 ± 259.1	554.2 ± 227.0	Intercept: 442.8 Slope: 38.5 (p < 0.001)
**South Asia**							
	**Female**	1296.1 ± 408.9	1205.3 ± 328.6	1196.0 ± 267.4	1372.9 ± 252.0	1793.7 ± 266.4	Intercept: 1105.6 Slope: 83.4 (p < 0.001)
	**Male**	242.8 ± 80.0	229.0 ± 68.9	228.3 ± 60.3	263.7 ± 59.3	296.7 ± 67.6	Intercept: 139.5 Slope: 48.4 (p < 0.001)
**South-East Asia, East Asia & Oceania**							
	**Female**	2023.6 ± 646.5	2130.0 ± 653.1	2136.2 ± 675.5	2163.7 ± 699.1	2117.4 ± 705.2	Intercept: 2085.5 Slope: 36.7 (p = 0.10)
	**Male**	472.6 ± 152.3	498.0 ± 154.2	504.6 ± 158.5	526.3 ± 179.3	533.5 ± 206.0	Intercept: 482.9 Slope: 19.2 (p < 0.001)
**Sub-Saharan Africa**							
	**Female**	2263.1 ± 777.1	2187.4 ± 811.2	2172.2 ± 883.5	2186.5 ± 943.8	2467.1 ± 913.6	Intercept: 2161.5 Slope: 24.4 (p = 0.32)
	**Male**	695.0 ± 353.2	693.9 ± 375.2	709.4 ± 403.4	723.5 ± 401.6	812.4 ± 423.1	Intercept: 676.9 Slope: 19.2 (p = 0.01)
Data shown as Mean ± SD. The intercepts assess the estimated overall mean level of secondary infertility rate in 1993. The slopes are interpreted as the average change in secondary infertility rate over time. GBD: Global burden of disease, LGM: Latent growth method							

## 4. Discussion

Infertility complications have been a concern across recent decades and are also a significant clinical problem today. In the present study, the infertility rate trend was explored in seven geographic regions designated by the GBD study.

The results demonstrated that during 1993-2017, the rate of primary infertility among men and women in High-Income countries declined. Although this trend also declined among Latin American and Caribbean men, this was not statistically significant. Other studies have claimed that male infertility has increased in developed countries and has become a problem worldwide during past decades (11). In the present study, it was observed that secondary male infertility increased over the time period, but primary male infertility decreased in the developed countries. In addition, the results showed that secondary infertility also decreased among women in Eastern and Central Europe and Central Asia, as well as in High-Income regions.

However, some studies have claimed that Central and Eastern European and Central Asian countries have high infertility rates, but this study suggested that infertility rates in these countries were lower than rates in African and other Asian countries (12).

The reason for the decrease of infertility in these regions may be that Europe is the continent with the lowest total fertility rate (13). In other words, because the tendency for having children is low, infertility remains unrecognized. In fact, it seems that there is a straight relationship between infertility and the fertility rate. Although this seems to be a contradiction, it may be because by reducing fertility among communities, many cases of infertility remain unknown. Some factors lead to lower fertility, including the instability of modern partnerships and value changes in these countries. A notable point is that the United States offers significant services to people in the treatment of infertility (14, 15).

Among other regions, both men and women in the Middle East and North Africa and also in South Asia have had the highest trend in rates of primary and secondary infertility in recent years. These results are in line with a systematic review related to increasing infertility in Africa (16). Various factors may be responsible for high infertility in the mentioned regions; in these regions, compared with the High-Income and other European countries, couples who already have a child go on to experience secondary infertility. Therefore, secondary infertility may be more commonly diagnosed in these countries, and the prevalence may be higher. Also, regions with a high prevalence of infectious diseases like HIV and also less industrialized nations have markedly higher infertility rates (17, 18).

The results indicated that the increase in infertility in developing countries was higher than in the developed ones. Various factors may cause a higher increase in infertility rates in these countries. Some of the factors that have been linked to an increase of infertility are dietary insufficiencies of iodine and selenium, and environmental and work-related toxicants including heavy metals, pesticides, arsenic, solvents, industrial chemicals and lead (19-22). Moreover, infertility treatment facilities in low- and middle-income countries are usually available only to wealthy people, so few have access to them (23, 24). Such populations tend to be conservative regarding things like sexuality, infertility and infectious diseases such as HIV. The silence around these issues contributes to accessibility disparities (25). This emphasizes the importance of infertility care availability and the socio-cultural value of childlessness and procreation in developing countries. Currently, there are differences between developed and developing populations in terms of these factors.

The other considerable finding in this research was that, not only was the growing trend of infertility in males estimated as lower than that of females, but also, women have had higher rates of infertility than men. In particular, in all regions, secondary infertility rates were several times higher in women than in men. In some studies, Sub-Saharan Africa has been named as the region with the highest prevalence of infertility (24). In this study, although the number of cases was high in women in Sub-Saharan Africa, the rate of growth was higher in the other regions, except for the High-Income and European and Central Asia regions. Female obesity is an increasing problem among some poor urban populations in developing countries, which is a risk factor for infertility in these countries (3). However, it is important to note that childbearing is considered a woman's duty in many countries, and men's information might not be collected. Infertility studies are generally performed on women (26). Also, the gold standard used for determining infertility in men makes it impossible to estimate the epidemiology of male infertility accurately (27).

One limitation of this study is that accurate and reliable information about the infertility rate does not exist for some countries. In developing countries, the infertility rates may be more accurate compared to in developed ones. In some populations, infertility is a less-discussed topic where getting support seems difficult, and due to the lack of information, there is a sense of shame tied to fertility challenges; hence, this may be the reason that infertility has not been explored and recorded correctly in these communities.

## 5. Conclusion

Although the infertility rate is reported differently worldwide, the overall trend of infertility prevalence shows a downward fashion in high-income and developed countries and an upward trend in other regions. This may be because in these regions there may be unrecognized infertility due to a low tendency for reproduction and because these regions have more infertility treatment facilities compared to the others. Clarification of the causes of these findings calls for more epidemiological studies.

##  Conflict of Interest

The authors declare that there is no conflict of interest.
